# Renal Venous Pattern: A New Parameter for Predicting Prognosis in Heart Failure Outpatients

**DOI:** 10.3390/jcdd5040052

**Published:** 2018-11-03

**Authors:** Agata Puzzovivo, Francesco Monitillo, Pietro Guida, Marta Leone, Caterina Rizzo, Dario Grande, Marco Matteo Ciccone, Massimo Iacoviello

**Affiliations:** 1 Cardiology Unit, IRCCS istituto tumori “Giovanni Paolo II” di Bari, Italia; agatapuzzovivo@yahoo.it; 2 Emergency Cardiology Unit, University Policlinic Hospital, 70124 Bari, Italy; fmonitillo@libero.it; 3 Cardiovascular Department, Scientific Clinical Institutes Maugeri, Institute of Cassano delle Murge, 70124 Bari, Italy; pieroguida@libero.it (P.G.); k.rizzo@live.it (C.R.); 4 Cardiology Unit, SS Annunziata Hospital, 74123 Taranto, Italy; martaleo84@yahoo.it; 5 School of Cardiology, University of Bari, 70124 Bari, Italy; dario.grande@ymail.com (D.G.); marcomatteo.ciccone@uniba.it (M.M.C.); 6 University Cardiology Unit, Cardiothoracic Department, Policlinic University Hospital, Piazza Giulio Cesare 11, 70124 Bari, Italy

**Keywords:** cardiorenal syndrome, prognosis, heart failure, renal Doppler, congestion

## Abstract

Aim of the study: In chronic heart failure (CHF) patients, renal congestion plays a key role in determining the progression of renal dysfunction and a worse prognosis. The aim of this study was to define the role of Doppler venous patterns reflecting renal congestion that predict heart failure progression. Methods: We enrolled outpatients affected by CHF, in stable clinical conditions and in conventional therapy. All patients underwent a clinical evaluation, routine chemistry, an echocardiogram and a renal echo-Doppler. Pulsed Doppler flow recording was performed at the level of interlobular renal right veins in the tele-expiratory phase. The venous flow patterns were divided into five groups according to the fluctuations of the flow. Type A and B were characterized by a continuous flow, whereas type C was characterized by a short interruption or reversal flow during the end-diastolic or protosystolic phase. Type D and E were characterized by a wide interruption and/or reversal flow. The occurrence of death and/or of heart transplantation and/or of hospitalization due to heart failure worsening was considered an event during follow-up. Results: During a median follow-up of 38 months, 126 patients experienced the considered end-point. Venous pattern C (HR 4.04; 95% CI: 2.14–7.65; *p* < 0.001), pattern D (HR 7.16; 95% CI: 3.69–13.9; *p* < 0.001) and pattern E (HR 8.94; 95% CI: 4.65–17.2; *p* < 0.001) were all associated with events using an univariate Cox regression analysis. Moreover, both the presence of pattern C (HR: 1.79; 95% CI: 1.09–2.97; *p*: 0) and of pattern D or E (HR: 1.90; 95% CI: 1.16–3.12; *p*: 0.011) remained significantly associated to events using a multivariate Cox regression analysis after correction for a reference model with an improvement of the overall net reclassification index (0.46; 95% CI 0.24–0.68; *p* < 0.001). Conclusions: Our findings demonstrate the independent and incremental role of Doppler venous patterns reflecting renal congestion in predicting HF progression among CHF patients, thus suggesting its possible utility in daily clinical practice to better characterize patients with cardio-renal syndrome.

## 1. Introduction

Over the last few decades, there has been a growing interest in the role of renal impairment in determining a worse prognosis of patients affected by chronic heart failure (CHF) [1,2,3]. In this setting it has been suggested that renal Doppler ultrasonography could have a role in detecting renal flow impairment and to better characterize renal function [4,5,6,7].

Using this technique, it is possible to evaluate not only the arterial flow and the arterial renal resistance, but also the renal venous blood flow [8,9,10,11]. In a population of outpatients and hospitalized heart failure patients, it has been recently demonstrated that the presence of an intra-renal intermittent venous flow with a biphasic or monophasic pattern [10] is associated with high central venous pressure, renal congestion and a worse prognosis. However, in this study, no data has been reported regarding the pattern observed in pre-eclamptic patients in which a reversal flow correspondent to right atrial contraction has been observed. This pattern seems to be between those showing a continuous and intermittent monophasic flow and is associated with an increased renal venous impedance index [11].

The aim of this study was to better define the role of all the possible renal Doppler venous patterns in predicting a worse prognosis in a large group of CHF outpatients in stable clinical conditions.

## 2. Methods

A population of consecutive outpatients affected by CHF of any origin and referred to the Heart Failure Unit of the Cardiology Unit of Policlinic Hospital of Bari were enrolled between 2013 and 2017. They were clinically stable for at least 30 days and had been receiving conventional medical therapy for at least 3 months if not contraindicated. Patients with acute worsening of kidney function or renal failure requiring dialysis were excluded from the study. All subjects gave their informed consent for inclusion before they participated in the study. The study was conducted in accordance with the Declaration of Helsinki, and the protocol was approved by the Ethics Committee of the Policlinic University Hospital of Bari (Project identification code: 4282).

### 2.1. Baseline Evaluations

At the time of enrollment a documented record of ischemic heart disease, arterial hypertension and diabetes mellitus was made for each patient. New York Heart Association (NYHA) class and arterial pressure were also evaluated. Heart rhythm and heart rate were assessed using 12-lead ECG.

Through echocardiographic evaluation (Vivid 7, GE Vingmed Ultrasound, General Electric, Milwaukee, WI, USA), the left ventricular ejection fraction (LVEF) was calculated using Simpson’s rule. E/e’ was calculated as the ratio between the early peak of E wave (E) at mitral pulsed Doppler and the mean of early peak of septal and lateral mitral annulus at tissue Doppler imaging (e’). Right ventricular systolic function was evaluated by calculating the systolic peak of tricuspid annulus excursion (TAPSE). Mitral (MR) and tricuspid regurgitation (TR) were semi quantitatively evaluated with arbitrary units ranging from 0 to 4.

Dilatation of the inferior vena cava and its collapsibility during inspiration were evaluated in order to assess central venous pressure (CVP) [12]. If inferior cava vein diameter was <2.1 cm and it collapsed >50% with a sniff, a CVP of 3 mm Hg (range, 0–5 mm Hg) was estimated. In presence of an inferior cava vein >2.1 cm which collapsed <50% with a sniff, a CVP of 15 mm Hg (range, 10–20 mm Hg) was considered. In presence of an inferior cava vein <2.1 cm, but with a collapse <50%, CVP was estimated as an intermediate value of 8 mm Hg (range, 5–10 mm Hg).

**Figure 1:**
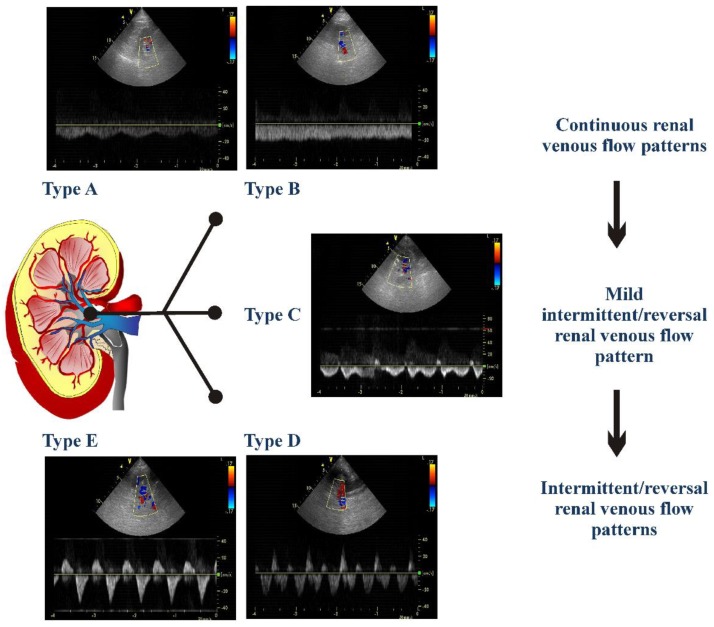
The different detectable renal venous patterns are presented. Pattern A was a flow pattern with normal velocity decrease of presystolic flow and biphasic pattern without interruption of telediastolic flow; Pattern B a continuous flow pattern with minimal fluctuations; Pattern C a pattern showing a short telediastolic interruption of forward flow or a short telediastolic reversal flow; Pattern D a flow characterized by a biphasic interruption or reversal flow during the same cardiac cycle; Pattern E a flow characterized by one forward and one reversal wave flow, i.e., monophasic intermittent pattern.

Renal Doppler was performed after echocardiographic examination using the same echocardiograph and probe (4 MHz). The patient was moved into a sitting position and a posterior approach to the kidney was used. In order to obtain the renal arterial resistance index (RRI), the course of right or left kidney segmental arteries were visualized by color Doppler flow. Then at the middle tract level of the best one visualized, a pulsed Doppler was performed. Every effort was made in order to reach the best alignment of the ultrasonic beam. An average of 2 to 3 measurements of peak systolic velocity and end diastolic velocity were used to calculate RRI according to Peurcelot’s formula, i.e., 100 × [1 − (end diastolic velocity/peak systolic velocity)] [6,13]. Venous Doppler was recorded at end-expiration. The analysis of venous patterns was performed by two observers (A.P., F.M.) who were blinded from patients’ follow-up. We decided to classify the patterns based on previous papers [8,9,10] but propose a new type of classification. As shown in Figure 1, the following venous patterns were identified for each patient: Pattern A including flow patterns with normal velocity decrease of presystolic flow and biphasic pattern without interruption of telediastolic flow; Pattern B including patterns of continuous flow with minimal fluctuations; Pattern C including patterns showing a short telediastolic interruption of forward flow or a short telediastolic reversal flow; Pattern D including patterns characterized by a biphasic interruption or reversal flow during the same cardiac cycle; Pattern E characterized by one forward and one reversal wave flow, i.e., monophasic intermittent pattern.

Blood samples were taken in order to assess serum creatinine, hemoglobin and NT-proBNP. The GFR was calculated using the Chronic Kidney Disease Epidemiology Collaboration equation (GFRCKD-EPI) [14]. By urine sample, normalbuminuria, microalbuminuria and macroalbuminuria were defined as the urinary albumin/creatinine ratio (UACR) of <30, 30 to 299, and ≥300 mg/g.

### 2.2. Follow-Up

The patients received follow-up as outpatients according to the protocol of our Heart Failure Unit. Information about deaths and hospitalizations was collected to reflect on the underlying cause. The primary end-point was considered as the occurrence of death and/or heart transplantation due to heart failure worsening and/or unplanned hospitalizations due to acute decompensated heart failure (ADHF). The timing to the first event was considered for the analysis.

### 2.3. Statistical Analysis

Continuous variables are expressed as mean values ± SD. In 20 randomly selected patients, the renal venous pattern was evaluated twice by the first observer and then by a second observer. The second observer was blinded to the evaluation of the first observer. Intra- and interobserver reproducibility was evaluated as a percentage of concordance of pattern definition.

Categorical variables are expressed as absolute frequency or percentage. Comparisons among groups were performed using an ANOVA analysis whilst Newman-Keuls tests were used for post-hoc comparisons. Comparisons among groups of the categorical variables were performed with a chi-square test or Fisher test.

The event-free survival curves were based on Kaplan–Meier analyses. Univariate Cox’s proportional hazards model was used to assess the association of variables with the endpoints. The reference model for the primary endpoint was developed with a Cox multivariate regression analysis. This included all univariate predictors by using a forward stepwise selection approach with *p* < 0.05 to retain covariates in the model. To avoid multicollinearity, redundant variables were dropped from the multivariable regression models. In the case of pairwise correlations between continuous variables exceeding 0.50 in Pearson’s coefficient, the variable with the strongest individual effect size was included [15]. Finally, to test the independent association of venous Doppler renal variables with the primary endpoint, they were analyzed in a Cox multivariate regression analysis with all the variables of the reference model. The effect of adding the renal venous pattern to the reference model on risk classification was evaluated with the use of category-free net reclassification improvement (NRI) [16]. The index quantifies improvement in the prediction of a risk factor added to a reference model by evaluating any upward or downward movement in predicted risks. A valuable new risk factor tends to increase predicted risks for events and decrease risks for nonevents.

Finally, in order to demonstrate that the renal venous Doppler parameters were associated with the events independently, a multivariate Cox regression model including microalbuminuria, GFR-EPI, RRI and renal venous patterns was analyzed.

The analyses were carried out using Statistica software, version 6.1 (StatSoft Inc., Tulsa, Oklahoma) and STATA software, Version 12 (StataCorp, College Station, TX, USA). *p*-values of <0.05 were considered statistically significant.

## 3. Results

Out of 343 patients, a total of 338 patients were enrolled. Two patients were excluded because their RRIs were not valuable and another three patients were in dialytic treatment. The remaining patients constituted our study population whose baseline clinical characteristics are shown in Table 1. Renal venous pattern was available in all the patients. All patients were in conventional therapy, 301 (89%) were previously implanted with a cardioverter defibrillator and 112 (33%) received cardiac resynchronization therapy. Sixty-one (18%) patients were in atrial fibrillation.

### 3.1. Reproducibility of Renal Venous Flow Pattern

The intra-operator concordance in the classification of renal venous flow pattern was 95%, whereas the interoperator concordance was 90%.

### 3.2. Clinical Correlates and Determinants of Renal Venous Flow Pattern

Table 1 shows the characteristics of patients according to the renal venous pattern. The major observed differences concern the patients of group D and E, which had a greater functional limitation, lower values of blood pressure, worse renal function, higher values of NT-proBNP, mitral and tricuspid regurgitation, central venous pressure, higher mean values of RRI and lower values of hemoglobin, LVEF, and TAPSE. Group D and E patients also showed a lower percentage of ACE inhibitors or ARBs therapy and a higher mean dose of furosemide. Compared to patients with pattern A or B, patients with pattern C, were characterized by greater values of NT-proBNP and right ventricular pressures. Interestingly, in presence of type D and E, 32 and 30% of patients, respectively, did not met echocardiographic criteria for high central venous pressure. In type C, this percentage was 70%.

### 3.3. Renal Venous Flow Pattern and Prognosis

During a mean follow-up of 35 ± 16 months (median 38 months, interquartile range 16–48 months), 78 patients died: 8 from sudden cardiac death, 54 because of worsening HF, 24 from other causes (6 due to non-cardiovascular causes). One hundred and twenty-two patients experienced at least one hospitalization due to ADHF and twenty-three underwent heart transplantation and/or left ventricular assist device. A total number of 126 patients experienced heart failure progression events.

Throughout univariate analysis, venous pattern C (HR 4.04; 95% CI: 2.14–7.65; *p* < 0.001), pattern D (HR 7.16; 95% CI: 3.69–13.9; *p* < 0.001) and pattern E (HR 8.94; 95% CI: 4.65–17.2; *p* < 0.001) showed a significantly greater probability of primary point events in comparison with Pattern A which was the reference group. No differences were found between pattern A and B and between pattern E and D, whereas both these pattern showed a greater probability of events when compared with pattern C. For the other analyses, we categorized three subgroups of patients: those with a continuous renal venous flow pattern (CRVP, i.e., patients showing pattern A and B), those with a mild intermittent/reversal renal venous flow pattern (MIRVP, i.e., patients showing pattern C) and those with an intermittent/reversal renal venous flow pattern (IRVP. i.e., patients showing pattern D and E). Table 2 shows the univariate predictors of events.

As shown in Table 3, in the stepwise forward Cox regression analysis, which included all the univariate predictors, NT-proBNP, NYHA class III, RRI > 75, heart rate > 70, MR, TAPSE ≤ 15 remained associated with the occurrence of primary end-point, but not age, diabetes, systolic arterial pressure, therapy with ACE-inhibitors/Angiotensin receptor blockers, therapy with beta-blockers, LVEF < 30%, tricuspid regurgitation, CVP > 5 mm Hg, GFR-EPI, presence of micro- or macroalbuminuria, hemoglobin.

In the multivariate Cox regression model including all the variables of the reference model both the presence of a MIRVP, i.e., Pattern C, and the presence of a IRVP, i.e., Pattern D or E, remained significantly associated with the events as well as RRI > 75 (Table 3). Moreover, the addition to the model including these parameters of MIRVP and IRVP improved reclassification according to NRI (0.46; 95% CI 0.24–0.68; *p* < 0.001).

When the presence of Pattern C and of Pattern D or E were added in a multivariate regression model including the other variables related with renal function, i.e., RRI, GFR-EPI and presence of micro- or macroalbuminuria, both remained significantly associated with events (HR: 2.24; 95% CI: 1.43–3.51; *p* < 0.001 and HR: 3.55; 95% CI: 2.30–5.47; *p* < 0.001, respectively).

**Figure 2:**
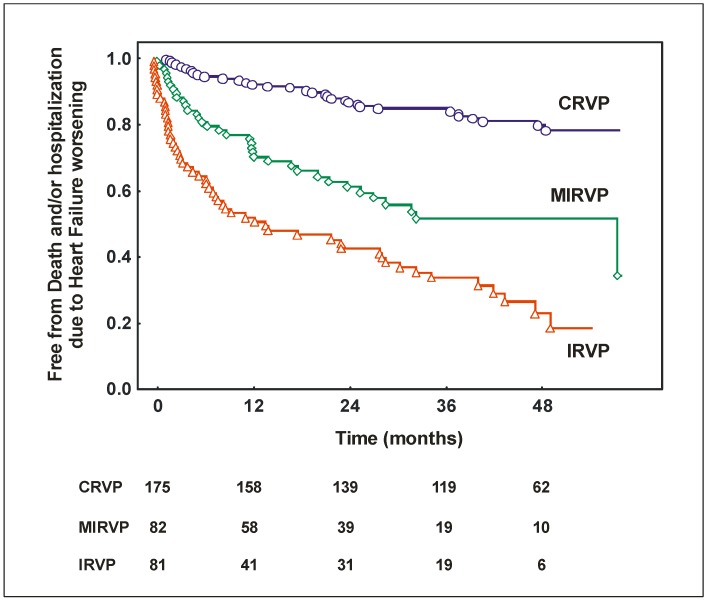
Kaplan Meier curves for the occurrence of death and/or hospitalization due to heart failure worsening in patients with continuous renal venous flow pattern (CRVP, i.e., Pattern A or B), mild intermittent/reversal renal venous flow pattern (MIRVP, i.e., Pattern C) and intermittent/reversal renal venous flow pattern (IRVP, i.e., Pattern D or E).

Figure 2 shows Kaplan Meier curves for the occurrence of death/hospitalization due to heart failure worsening and for the occurrence of death for all causes in patients with CRVP, MIRVP and IRVP. 

## 4. Discussion

The main finding of this study is that the renal Doppler venous patterns characterized by an intermittent or reversal flow show an independent and incremental role in predicting a worse prognosis of CHF outpatients. The predictive value of renal venous patterns seems to be related to its ability to better detect renal venous congestion. This strengthens the possible role of Doppler ultrasonography in daily clinical practice in order to better characterize the relationship between cardiac and renal dysfunction.

In CHF patients, renal function impairment and its worsening [1,2,3,17] are frequently observed conditions which are independently associated with greater morbidity and mortality. This prognostic impact is due to the complex pathophysiological link that exists between the heart and the kidney. This has recently been defined as cardiorenal syndrome, i.e., the condition characterized by an acute or chronic dysfunction of one of the two organs which may induce the acute or chronic dysfunction of the other [2].

Among the pathophysiologic factors responsible for the progression of cardiorenal syndrome, the abnormalities in renal perfusion can play a key role [18]. An abnormal renal blood flow can be determined either by hypoperfusion related to low cardiac output or by congestion related to increased CVP [19]. The role of increased CVP in determining the progression of cardio-renal syndrome in both acute decompensated heart failure and chronic heart failure has been widely demonstrated [19,20]. High values of central venous pressure can be transmitted to the renal veins causing an increase in efferent arteriolar and glomerular capillary pressure, and consequently a reduction of the net filtration pressure and of the glomerular filtration rate [21].

In daily clinical practice, the estimation of CVP through the measurement of the inferior vena cava diameter [11] is the only echographic parameter that is routinely assessed and can be useful to detect the presence of renal congestion in heart failure patients. In this clinical setting the study of renal venous flow by the Doppler technique can improve the ability in detecting the presence of an increased renal venous pressure and, as a consequence, the presence of renal congestion. In presence of high right atrial pressures, the renal venous flow pulsatility can be altered and renal blood flow can be stopped or reversed. In a recent study by Iida et al. [10], the presence of an intermittent renal venous flow has been demonstrated to be strongly related with invasively measured high central venous pressure and it has been associated with a worse prognosis.

Our findings confirm these results but also offer further data supporting the possible usefulness of the study of renal venous flow in order to better detect renal congestion and to stratify prognosis of CHF outpatients. Like in the study of Iida and colleagues, in our series, the presence of intermittent venous patterns, that we defined as D or E, is associated with an increased estimated central venous pressure. However, it is worth noting that around 30% of patients showing these patterns do not present increased CVP assessed by inferior cava vein diameter. Moreover, the prognostic value of these patterns was independent and incremental from that of high CVP. As a consequence, these renal venous Doppler patterns seem to reflect more accurately than the estimated CVP of renal congestion.

Beyond the prognostic role of IRVP, i.e., patterns D and E, our data also demonstrate that another pattern, that we defined as MIRVP or pattern C, is independently associated with a worse prognosis. Although IRVP is poorly correlated with the presence of high CVP. This type of pattern has been previously described in pre-eclamptic patients [9] and it is characterized by an end-diastolic or proto-systolic interruption of the flow that can be associated to the presence of a reversal flow. Pattern C can be the expression of a mild increase of central venous pressure, which becomes evident when a further increase of right atrial pressure is transmitted to renal veins and it is able to stop or reverse the blood flow. In particular, the right atrium pressure changes along the cardiac cycle which is responsible for the triphasic modulation of venous blood flow at the level of the inferior vena cava and hepatic veins. The transient increase of atrial pressure during atrial systole and/or the closure of tricuspid leaflets at the beginning of systole can cause an interrupted or a reversal flow. This hypothesis is supported by a recent study which evaluated the effect of volume expansion on intrarenal venous flow [11]. When the atrial pressures further increase, the interruption of the flow is longer and the intermittent patterns D and E can be observed. On the basis of these considerations, the presence of a mild intermittent/reversal renal flow pattern can detect early renal venous blood flow abnormalities related to congestion, thus also explaining its independent and incremental role in predicting a worse prognosis.

Although our findings about renal venous patters are similar to those recently published by Iida and colleagues [10], there are several differences. First of all, we enrolled a larger series of patients who were in stable clinical conditions and who were followed-up over a longer period. Secondly, we identified the pattern C as associated with a mild renal congestion and worse prognosis when compared with continuous renal flow patterns, i.e., A and B. Thirdly, we have demonstrated that the evaluation of venous flow pattern can integrate the information obtained from the assessment of RRI in order to identify patients more prone to experience worsening heart failure. Arterial and venous renal Doppler are able to offer different and complementary information. In fact, arterial Doppler and RRI can more narrowly reflect vascular and parenchymal renal abnormalities as well as neurohormonal hyperactivity and congestion related to high intrarenal pressures. Whereas renal venous patterns can more accurately reflect the presence of renal congestion. The independent association with the events of renal venous patterns and RRI supports this hypothesis. Finally, the relevant clinical role of arterial and venous Doppler ultrasonography is further supported by the fact that their prognostic value, in our series, is independent from the estimation of GFR and microalbuminuria which are the renal function parameters with a proven prognostic role in CHF outpatients [1,22].

Limitations of the study: This is an observational study which needs to be confirmed by larger multicenter studies. Although we demonstrated the prognostic relevance of renal venous Doppler pattern, GFR remains the main parameter for the assessment of renal function in type 2 cardiorenal syndrome. Renal venous Doppler pattern could provide a further characterization of renal function but it cannot be proposed in routine clinical practice. Further studies should confirm the independent prognostic value of renal venous patterns as well as evaluate if there is a relationship among changes in renal venous patterns, changes in the other parameters reflecting congestion and its relationship with a worse prognosis. This could be useful in order to understand if renal venous patterns can be proposed not only as marker of prognosis but also for tailoring patients’ therapy in order to reduce the negative effects of renal congestion. Finally, although the evaluation of the renal venous pattern can be easily obtained at the end of a standard echocardiographic examination, future studies should also demonstrate the cost effectiveness of this examination in heart failure patients.

In conclusion, renal venous Doppler patterns reflecting renal congestion show an independent and incremental role in predicting a worse outcome in CHF outpatients. These results suggest the possible clinical usefulness of renal venous Doppler evaluations in better characterizing cardio-renal syndrome. Therefore, the present findings may represent the basis for further studies in larger samples aimed at defining the possible use of the renal venous Doppler in daily clinical practice, as well as the possibility of using it for a tailored therapeutic approach.

## Figures and Tables

**Table 1:** Baseline clinical characteristics of all patients and of patients divided according to the venous pattern.

	All Patients			Pattern			
		A	B	C	D	E	p
	n: 338	n: 83	n: 92	n: 82	n: 41	n: 40	
Age (years)	64 ± 13	62 ± 14	61 ± 13	66 ± 13	66 ± 14	67 ± 12	0.016
Males (%)	81	83	84	85	76	70	0.221
Ischemic cardiomyopathy (%)	42	47	43	40	49	40	0.459
History of hypertension (%)	60	65	49	66	63	57	0.133
Diabetes mellitus (%)	30	33	25	33	27	35	0.673
NYHA class	2.4 ± 0.6	2.2 ± 0.6	2.2 ± 0.6	2.4 ± 0.5	2.6 ± 0.5 *^,†,‡^	2.8 ± 0.4 *^,†,‡^	<0.001
SBP (mm Hg)	122 ± 16	126 ± 15	121 ± 14	124 ± 17	120 ± 17	113 ± 17 *^,†,‡,#^	<0.001
Heart rate (beats per minute)	69 ± 11	67 ± 9	67 ± 8	70 ± 13	71 ± 11	73 ± 10 ^†,‡^	0.002
GFR-EPI (mL/min/1.73 m^2^)	72 ± 26	79 ± 23	79 ± 25	70 ± 25	63 ± 30 *^,†^	57 ± 22 *^,†,‡^	<0.001
Hb (g/dL)	13.6 ± 1.6	13.9 ± 1.5	14.0 ± 1.4	13.9 ± 1.6	12.6 ± 1.6 *^,†,‡^	12.6 ± 1.6 *^,†,‡^	<0.001
NT-proBNP (pg/mL)	2311 ± 4844	827 ± 818	1171 ± 1490	2330 ± 2492 *^,†^	4313 ± 3760 *^,†,‡^	5884 ± 11981 *^,†,‡^	<0.001
Microalbuminuria (%)	27	18	16	28	61 *^,†,‡^	42 *^,†^	<0.001
Ace-inhibitors/ARBs (%)	81	93	88	74 *	76 *	60 *^,†^	<0.001
Beta-blockers (%)	97	99	98	98	98	88 *^,†,‡^	0.013
Diuretics (%)	93	88	88	96 *^,†^	100 *^,†^	100 *^,†^	<0.001
Furosemide dose (mg/die)	106 ± 131	68 ± 76	70 ± 72	111 ± 137	146 ± 179 *^,†^	193 ± 176 *^,†,‡,#^	<0.001
MRAs (%)	72	76	71	71	66	87	0.110
LVEF (%)	33 ± 9	36 ± 7	33 ± 8	32 ± 8	29 ± 11 *	31 ± 13 *^,†^	<0.001
E/e’ ratio §§	14.8 ± 7.4	13.0 ± 6.0	12.1 ± 5.4	14.4 ± 6.2	14.3 ± 8.8	17.9 ± 7.4 *^,†,‡,#^	0.014
TAPSE (mm)	19 ± 4	20 ± 4	19 ± 4	19 ± 5	17 ± 4 *^,†,‡^	16 ± 4 *^,†,‡^	<0.001
MR (a.u.)	1.8 ± 0.9	1.5 ± 0.8	1.6 ± 0.8	1.8 ± 0.9	2.2 ± 0.9 *^,†,‡^	2.1 ± 1.1 *^,†^	<0.001
TR (a.u.)	1.8 ± 0.9	1.5 ± 0.7	1.4 ± 0.7	1.5 ± 0.7	2.7 ± 1.1 *^,†,‡^	2.8 ± 1.2 *^,†,‡^	<0.001
CVP > 5 mm Hg (%)	26	5	2	30 *^,†^	68 *^,†,‡^	70 *^,†,‡^	<0.001
RRI	73 ± 8	71 ± 7	70 ± 7	74 ± 8 *^,†^	78 ± 8 *^,†,‡^	77 ± 7 *^,†,‡^	<0.001

* *p* < 0.05 vs. pattern A; ^†^ vs. pattern B; ^‡^ vs. Pattern C; **^#^** vs. Pattern D at Newman-Keuls post-hoc analysis or Fisher test as appropriate. § Analysis for 307 patients without macroalbuminuria. §§ Available in 261 patients. Mean values ± SD or Percentage of patients. The table shoes ANOVA test for continuous variables F-Fisher tests for categorical variables. For NT-proBNP analyses were performed after logarithmic transformation. ARBs: Angiotensin II receptor blockers; a.u., arbitrary units; CVP, central venous pressure; GFR-EPI, glomerular filtration rate by EPI formula; MRA: Aldosteron receptor antagonist; LVEF, left ventricular ejection fraction; MR, mitral regurgitation; NYHA, New York Heart Association; NT-proBNP: brain natriuretic peptide. RRI, renal resistance index; TAPSE, peak of tricuspid annular plane systolic excursion; TR, tricuspid regurgitation.

**Table 2:** Univariate analysis for primary end-point. For abbreviations see Table 1.

	Cox Univariate Analysis	
	HR (95% CI)	*p*
Age	1.02 (1.01–1.04)	0.001
Diabetes	1.47 (1.03–2.12)	0.036
Ischemic aetiology	1.52 (1.07–2.15)	0.019
Systolic arterial pressure	0.98 (0.96–0.99)	<0.001
ACE-I and/or ARBs	0.33 (0.23–0.49)	<0.001
Beta-blocker	0.28 (0.14–0.57)	<0.001
NYHA class III	5.67 (3.82–8.39)	<0.001
Heart rate > 70	1.64 (1.15–2.35)	0.007
LVEF < 30%	3.27 (2.58–4.75)	<0.001
Mitral regurgitation	1.94 (1.62–2.33)	<0.001
TAPSE < 15	2.91 (2.03–4.16)	<0.001
Tricuspid regurgitation	1.87 (1.59–2.21)	<0.001
CVP > 5 mm Hg	3.23 (2.26–4.64)	<0.001
GFR-EPI	0.98 (0.97–0.98)	<0.001
Micro- or macroalbuminuria	3.11 (2.19–4.43)	<0.001
Hemoglobin	0.74 (0.66–0.83)	<0.001
NT-proBNP (×100)	1.03 (1.02–1.03)	<0.001
RRI > 75	2.99 (2.07–4.33)	<0.001

**Table 3:** Reference model derived by using a forward stepwise Cox analysis where renal venous patterns categories have been included.

	Reference Model		Reference Model Plus Renal Venous Patterns	
	HR (95% CI)	*p*	HR (95% CI)	*p*
NYHA class III	3.01 (1.97–4.59)	<0.001	2.72 (1.76–4.20)	<0.001
Heart rate > 70	1.69 (1.17–2.44)	0.005	1.55 (1.07–2.27)	0.005
Mitral regurgitation	1.29 (1.07–1.56)	0.008	1.25 (1.04–1.52)	0.008
TAPSE < 15	1.59 (1.09–2.32)	0.017	1.53 (1.05–2.25)	0.017
NT-proBNP (×100)	1.020 (1.015–1.026)	<0.001	1.02 (1.012–1.023)	<0.001
RRI > 75	1.92 (1.31–2.81)	0.001	1.69 (1.14–2.50)	0.001
CRVP (type A or B)			1.00	-
MIRVP (type C)			1.79 (1.09–2.97)	0.023
IRVP (type D or E)			1.90 (1.16–3.12)	0.011

CRVP: continuous renal venous flow pattern. IRVP: intermittent/reversal renal venous flow pattern. MIRVP: mild intermittent/reversal renal venous flow pattern. For the other abbreviations see Table 1.
